# Three-Dimensional (3D)-Printed Snacks from Indigenous Composite Inks Improve Metabolic Dysfunctions Associated with High-Fat-Diet-Induced Obesity in Wistar Rats

**DOI:** 10.3390/foods14244185

**Published:** 2025-12-05

**Authors:** Abdullahi Adekilekun Jimoh, Abidemi Paul Kappo, Fehintoluwa Joy Femi-Olabisi, Yusuf Olamide Kewuyemi, Omolola Mary Omosebi, Oluwafemi Ayodeji Adebo

**Affiliations:** 1 Centre for Innovative Food Research (CIFR), Department of Biotechnology and Food Technology, Faculty of Science, University of Johannesburg, Doornfontein, Johannesburg 2094, South Africa; jimohabdullahi1@gmail.com (A.A.J.); yusufkewuyemi@gmail.com (Y.O.K.); 2 Molecular Biophysics and Structural Biology Group, Department of Biochemistry, Faculty of Science, University of Johannesburg, Auckland Park, Johannesburg 2005, South Africa; akappo@uj.ac.za; 3 Department of Biochemistry, Mountain Top University, Prayer City 110106, Nigeria; 4 Department of Food Science and Technology, Mountain Top University, Prayer City 110106, Nigeria

**Keywords:** 3D printing, probiotic fermentation, short-term germination, composite bioprocessed inks, high-fat diet, obesity, Wistar rats

## Abstract

This study investigated the anti-obesogenic effects of 3D-printed snacks—developed from indigenous composite inks of cowpea, sorghum, and orange-fleshed sweet potato—in male and female Wistar rats fed a high-fat diet (HFD). Four experimental diets (TD1–TD4) were formulated from snacks using two blend ratios (33.33%:33.33%:33.33%) and 50%:10%:40%) and two processing states (raw and bioprocessed). Following a five-week HFD-induction period, the rats were supplemented for an additional five weeks with diets containing 20% of these snacks, Orlistat, or HFD alone. Physiological assessments included body weight, fasting glucose, insulin, homeostatic model assessment for insulin resistance (HOMA-IR), serum lipids, sex hormones, angiotensin-converting enzyme (ACE) activity, and histological evaluation of cardiac tissue. HFD feeding induced hyperglycemia, dyslipidemia, and insulin resistance. Supplementation with the 3D-printed snacks improved glycemic control, with the TD4 (bioprocessed blend; 50:10:40%) restoring glucose levels close to baseline. TD1 and TD2 (raw blends) improved lipid and hormonal profiles in females, whereas TD3 (bioprocessed blend; 33.33%:33.33%:33.33%) significantly reduced triglycerides and elevated HDL in males. Importantly, only TD1 (raw blend; 33.33%:33.33%:33.33%) significantly reduced ACE activity in males, providing a unique cardioprotective mechanism not observed with other snack formulations. Histological analyses revealed inflammatory infiltration and fibroplasia in HFD and Orlistat groups, whereas all 3D-printed snacks preserved normal myocardial architecture without necrosis or fibrosis. Collectively, these findings demonstrate that 3D-printed snacks derived from indigenous composite inks improved metabolic dysfunctions associated with diet-induced obesity. The optimal formulation appears application-specific: TD4 for glycemic control, TD3 for lipid management in males, and TD1/TD2 for metabolic improvements in females.

## 1. Introduction

Obesity is a complex disease characterized by the excessive accumulation of adipose tissue and has become a global public health concern affecting individuals across all age groups [1,2,3]. Sedentary lifestyles, high-calorie diets, and genetic predisposition are primary contributors to being overweight and obesity. If left untreated, these factors can lead to metabolic complications, such as elevated blood glucose, insulin resistance, dyslipidemia, and hypercholesterolemia [2,4]. Obesity is not only a condition in itself but a critical risk factor for several non-communicable diseases (NCDs), including type 2 diabetes mellitus, cardiovascular diseases, and inflammatory bowel disease [3,5]. According to the World Health Organization (WHO), an estimated 2.5 billion adults were overweight in 2022, with more than 890 million classified as obese. Conventional therapeutic strategies, such as lifestyle modification, bariatric surgery, and pharmacotherapy, have shown limited long-term success in managing obesity, underscoring the urgent need for innovative, sustainable alternative approaches to obesity management [6,7].

Diet-based interventions remain a cornerstone in the prevention and management of obesity, with functional foods gaining increasing attention for their ability to modulate metabolic health. Functional foods are defined as those that provide health benefits beyond basic nutrition, often through bioactive compounds that target specific physiological pathways. Plant-based functional foods rich in dietary fiber, phenolic compounds, bioactive peptides, and probiotics have shown potential in improving lipid metabolism, enhancing glycemic control, and reducing systemic inflammation [8,9].

Traditional food processing techniques, such as germination and fermentation, play a significant role in enhancing food composition and inducing beneficial biochemical transformations [10]. Germination activates endogenous enzymes that improve the release of vitamins, minerals, amino acids, and phenolic compounds, increase dietary fiber content, and reduce antinutritional factors [11]. Similarly, fermentation, particularly probiotic fermentation, introduces beneficial microbial metabolites, such as short-chain fatty acids, bioactive peptides, and exopolysaccharides, which are associated with improved gut health, anti-inflammatory activity, and metabolic regulation [12]. Composite flours from bioprocessed ingredients such as germinated cowpea and probiotic-fermented sorghum and orange-fleshed sweet potato (OFSP) can interact synergistically to enhance bioactive components, inhibit key digestive enzymes, and serve as functional ingredients suitable for developing novel foods [13]. These functional properties suggest that when used to develop foods such as three-dimensional (3D)-printed snacks, these composites may help attenuate obesity-related metabolic challenges.

3D food printing has emerged as an innovative technology in functional food design, offering precise control over composition, scalability, shape, and nutrient delivery [14]. Hot-extrusion 3D printing (HE-3DP), in particular, can modify food components to enhance their health-promoting potential [15,16]. This advanced processing tool enables the creation of snacks with tailored nutritional and functional properties by integrating bioprocessed flours [10]. Recent research highlights the anti-obesity potential of 3D-printed foods. Notably, Guo et al. [16] used HE-3DP to produce whole-grain barley pre-made biscuits with lower starch digestibility. Their subsequent in vivo study indicated that these biscuits could modulate glycolipid metabolism and ameliorate high-fat-diet-induced metabolic disorders in male rats. In another study, a low-calorie, 3D-printed citrus-based jelly exhibited anti-obesity and antioxidant properties, suppressing body weight gain and fat accumulation in male mice [17]. Other work has successfully incorporated modified okara fiber into 3D-printed cookies to increase their dietary fiber content and nutritional value [18]. However, these studies did not utilize traditional bioprocessing methods, such as germination or probiotic fermentation, in their formulations.

Despite the growing interest in 3D-printed functional foods from bioprocessed flours [10], little in vivo evidence exists regarding their efficacy against obesity-related outcomes and sex-specific effects. Therefore, this study investigated whether snacks formulated from germinated and probiotic-fermented flours, developed via HE-3DP, can reduce high-fat-diet (HFD)-induced metabolic dysfunction in male and female Wistar rats.

## 2. Materials and Methods

### 2.1. Materials

Raw materials were sourced from the Agricultural Research Council in Nelspruit, the Fish on Tackle Store in Kempton Park, and the Woolworths store in Johannesburg, South Africa. Other edible materials, including corn flour, defatted soybeans, salt, sucrose, and animal fat, were purchased from Ibafo market in Ibafo, Ogun State, Nigeria. Similarly, a CHN-22 freeze-dried starter culture containing *Lactococcus lactis* subsp. *cremoris*, *Lactococcus lactis* subsp. *lactis*, *Lactococcus lactis* subsp. *lactis* biovar. *diacetylactis*, *Leuconostoc mesenteroides*, and *Leuconostoc pseudomesenteroides* were obtained from Chr. Hansen Holding A/S, Hørsholm, Denmark.

Assay kits used in the study were supplied by Randox Laboratory (County Antrim, UK) and Roche Diagnostics GmbH (Mannheim, Germany). An Accu-Chek Active Compact Plus glucometer and test strips were used for blood glucose measurements. Orlistat (Ecoslim) was provided by Micro Labs Limited (Thiruvandarkoil, Puducherry, India), while angiotensin-converting enzyme (ACE) assay reagents were obtained from Fortress Diagnostics Limited (Antrim, Northern Ireland).

### 2.2. Formulation of Composite Flours for 3D Printing

The raw or bioprocessed composite flours were prepared following the detailed procedure reported by Kewuyemi and Adebo [11]. The obtained raw flours were from cowpea, sorghum, or orange-fleshed sweet potato (OFSP). The bioprocessed flours were germinated cowpea, probiotic fermented sorghum, and OFSP. Composite flours (either raw or bioprocessed flours) were prepared in two flour-to-flour ratios (*w*/*w*): (a) 33.33%:33.33%:33.33% and (b) 50%:10%:40%. The selected flour-to-flour ratio (50%:10%:40%) was chosen because it demonstrated a better, synergistic enhancement of phenolic compounds and the most potent in vitro inhibition of starch and lipid hydrolyzing enzymes compared to other ratios in our previous work [13]. This comparative approach enables a direct assessment of whether the functionally superior blend confers better anti-obesity benefits in vivo than a standard formulation. The composite flours were blended for 15 min using an electric mixer (KMM7XX, Kenwood, Watford, UK) with intermittent manual blending at five-minute intervals. The obtained raw composite flours were composite A (33.33% raw cowpea:33.33% raw sorghum:33.33% raw orange-fleshed sweet potato flours) and composite B (50% raw cowpea:10% raw sorghum:40% raw orange-fleshed sweet potato flours). The bioprocessed composite flours were composite C (33.33% germinated cowpea:33.33% fermented sorghum:33.33% fermented OFSP flours) and composite D (50% germinated cowpea:10% fermented sorghum:40% fermented OFSP flours).

### 2.3. 3D Printing and Post-Processing of Composite Snacks

**Figure 1:**
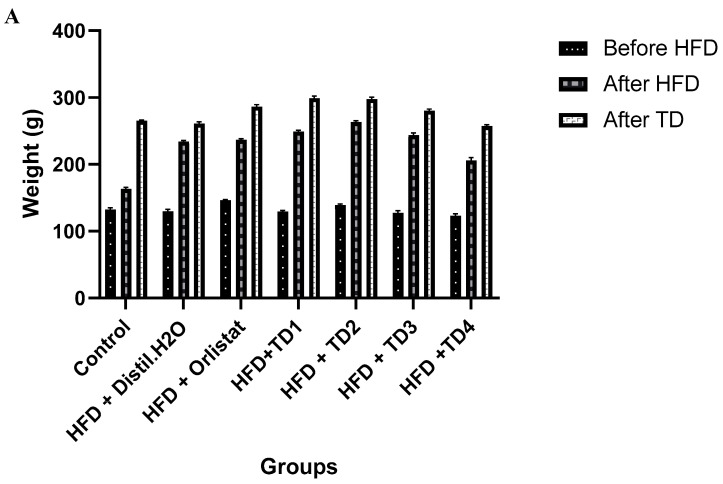

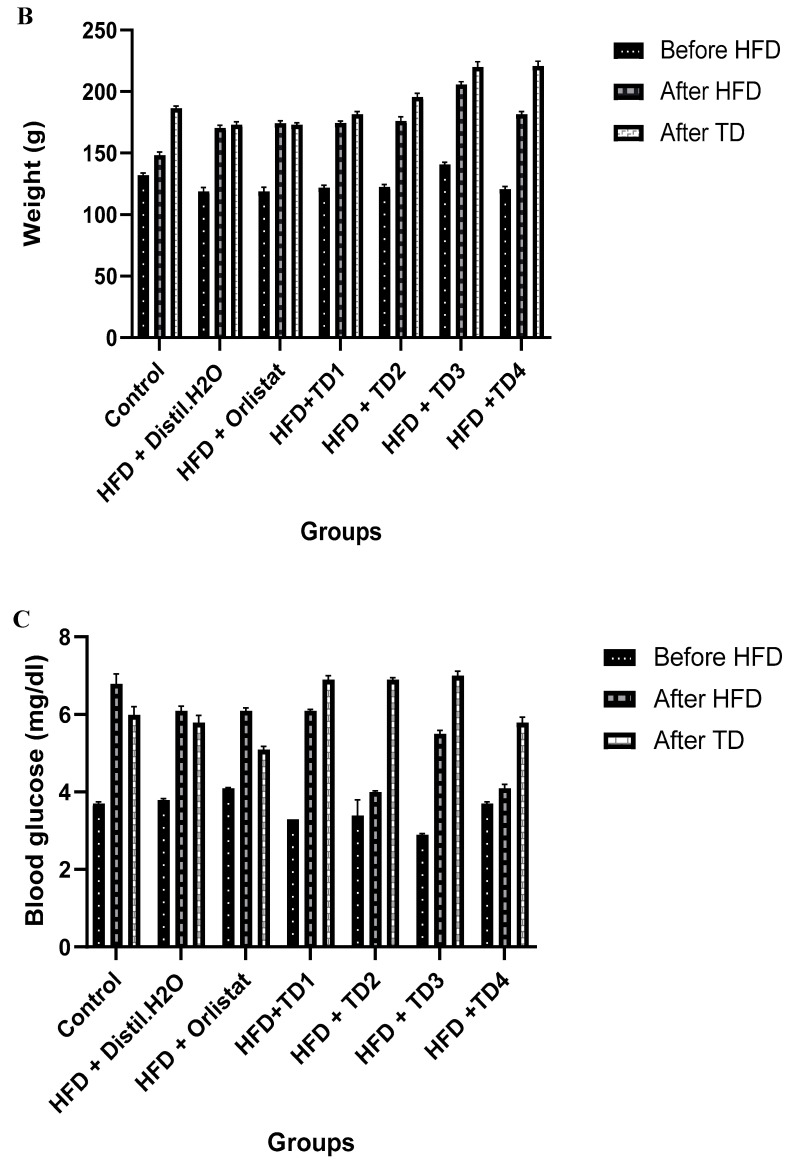

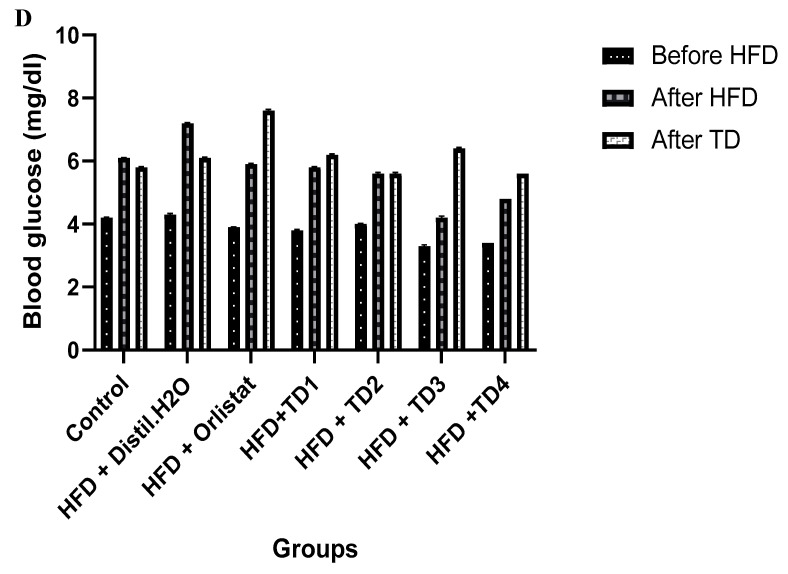
Effect of dietary interventions with HFD on (**A**) body weight of male rats, (**B**) body weight of the female rats, (**C**) blood glucose of male rats, (**D**) blood glucose of female rats. Values were expressed as mean ± SEM (*n* = 7).

Preliminary experiments determined that a mixture of 50 g composite flour (dry basis) and 50 g hot distilled water (75 °C) produced the best printability. Doughs (50:50 *w*/*w*) prepared from composite flours A–D were 3D printed using a Foodini 3D printer (Natural Machines, Barcelona, Spain). A snowflake-shaped model (diameter [53 mm], width [51 mm], and thickness [4 mm]) (Figure 1) was selected on Foodini Creator. The doughs were carefully loaded into the printer cartridge and printed (3 min 25 s) using pre-set printing conditions: print speed (2500 mm/min), line thickness (1.2 mm), nozzle size (1.5 mm), nozzle height (1.5 mm), pre-heat, and ingredient flow temperature (75 °C). The printed constructs were placed at −21 °C (KBF 631, KIC SA, Pty, Ltd., Johannesburg, South Africa) for two hours to maintain structural integrity [19]. Among various post-processing methods tested (air frying, baking, hot air drying, and microwave heating), microwave heating was selected due to its rapid volumetric heating mode and superior preservation of nutritional and health-promoting constituents. The 3D constructs were cooked in a pre-heated microwave (MM0001, Milex, Homemark, Pty, Ltd., Sandton, South Africa) using programmed firepower (9P, On 26s/Off 3s cycle) for 18 min. The snacks were transferred, cooled for one hour, and packed in airtight transparent food-grade mini bags.

### 2.4. In Vivo Study

#### 2.4.1. Experimental Animals and Study Design

The in vivo study for the use of Wistar rat model was approved by the Ethics Committee of the Faculty of Science, University of Johannesburg (Reference number: 2024-06-06/Kewuyemi_Adebo) and the Ethical Review Committee at Mountain Top University (Reference number: MTU–ERC–011/24). A total of 70 healthy male and female Wistar rats (6–8 weeks old, average weight: 180–220 g) were obtained from Mctemmy Concept Laboratory Animals, Ogbomoso, Oyo, Nigeria, and acclimatized for two weeks under controlled conditions in the Animal House of the Department of Biochemistry, Mountain Top University, with a 12 h light/12 h dark cycle, temperature of 22–24 °C, and relative humidity of 40–60%. The rats were maintained on a basal diet containing corn flour, defatted soybeans, salt, and sucrose. The animals were then randomly divided into seven experimental groups (*n* = 7 per group, with equal numbers of males and females), with males and females housed in separate cages. Group 1 was maintained on the basal diet throughout the 10-week experimental period, while Groups 2–7 were fed a high-fat diet (HFD) for the first 5 weeks. During the subsequent 5-week intervention phase, the HFD-fed groups received one of six dietary treatments: HFD + distilled water, HFD + orlistat, HFD + TD1, HFD + TD2, HFD + TD3, or HFD + TD4. The general health and conditions of the rats were monitored daily throughout the experiment.

#### 2.4.2. High-Fat and Test Diet Formulation

The HFD formulation consisted of a modified basal diet, which contained corn flour (357.5 g), defatted soybeans (335 g), salt (2.5 g), sucrose (100 g), and animal fat (205 g). The test diets were formulated by replacing 20% of the basal diet with 3D snacks. The test diets (TD) contained TD1 (a diet containing snacks from composite A), TD2 (a diet containing snacks from composite B), TD3 (a diet containing snacks from composite C), and TD4 (a diet containing snacks from composite D).

### 2.5. Analysis

Average body weight and fasting blood glucose (mg/dL) were measured at baseline, post-HFD induction, and post-intervention using an Accu-Chek glucometer (Roche Diagnostics, Mannheim, Germany).

For glucose and insulin assessment, animals were fasted for 12 h prior to tail vein bleeding into heparinized tubes. The glucose concentrations were determined using a glucometer (Accu-Chek Performa, Roche Diagnostics, Mannheim, Germany), while serum insulin levels were quantitatively determined using a microplate immunoassay kit as described in the manufacturer’s protocol [20]. The Homeostatic Model Assessment for Insulin Resistance (HOMA-IR) score was calculated using the following equation based on fasting insulin and glucose levels.

HOMA-IR = fasting glucose (mg/dL) × fasting insulin (μIU/mL)/22.5

The serum ACE level was quantitatively determined using an ACE kit (Fortress Diagnostics Limited, Belfast Road, Co. Antrim, Northern Ireland), as described in the manufacturer’s protocol.

Lipid levels analysis: At the end of the intervention trial, the animals were euthanized under isoflurane anesthesia, and blood was collected via jugular puncture. Serum was separated by centrifugation (4000× *g* for 10 min) and stored at −4 °C. Serum triglycerides (TG), total cholesterol (TC), high-density lipoprotein (HDL) cholesterol, and low-density lipoprotein (LDL) cholesterol were analyzed using Randox kits following the manufacturer’s instructions (Randox Laboratory Ltd., Crumlin, County Antrim, Northern Ireland).

The serum testosterone concentration was determined using testosterone ELISA Kits (PerkinElmer Laboratories, Freiburg, Germany) according to the manufacturer’s protocol. The estradiol concentration was measured using a direct immunoenzymatic approach with an ELISA kit also purchased from PerkinElmer Laboratories, Freiburg, Germany.

Histological analysis: The collected organ tissues fixed in 10% formalin were dehydrated in varying concentrations of ethanol solution (70%, 90%, and 95%, respectively). Following Krause’s [21] protocol, the organs were cleaned with xylene and formed into paraffin blocks. The tissues were prepared and stained using hematoxylin and eosin. A light microscope (CX21FS1, Olympus Scientific Solutions, Waltham, MA, USA) was used to read the processed histology slides, and the photomicrographs of the organs were acquired.

### 2.6. Statistical Analysis

The statistical differences were determined using one-way variance (ANOVA) and the Duncan multiple range test (IBM SPSS Statistics, Version 23, IBM Corp., Armonk, NY, USA). The data were presented as means ± standard error of the mean (SEM), and statistically significant values are denoted by *p* < 0.05.

## 3. Results

### 3.1. Changes in Body Weight and Fasting Blood Glucose of HFD-Fed Rats

Figure 1A,B illustrates the changes in body weight of rats throughout the experimental phases. Following HFD administration in both sexes, body weight significantly increased in all groups, confirming the successful induction of obesity. After the intervention period, male rats in the TD1–TD4 groups weighed between 257.42 ± 1.96 g and 299.25 ± 3.08 g, indicating sustained weight gain despite the treatment. In female rats, post-treatment body weights ranged from 181.82 ± 2.05 g in the HFD + Water group to 220.98 ± 3.70 g in the TD4 group. This suggests that the treatments may exert effects through mechanisms other than overall weight loss. Although a 10-week period was sufficient to induce obesity, it might not have been long enough to reverse established adiposity.

The fasting blood glucose results showed that the HFD successfully induced hyperglycemia in both sex groups, confirming diet-related disruption of glucose homeostasis and increased cardiometabolic risk (Figure 1C,D). Among the test diets, TD4 showed the most significant improvement in glycemic control. In males, fasting glucose levels decreased to 4.1 ± 0.1 mmol/L after TD4 treatment, compared to the control and HFD groups. This reduction was sustained post-treatment. Similarly, in females, TD4 lowered glucose levels following HFD and maintained lower values compared to the HFD + water group. By contrast, TD1–TD3 produced only partial reductions in glucose levels, and in male rats in these groups showed significant rebound hyperglycemia post-treatment (TD2 = 6.9 ± 0.05; TD3 = 7.0 ± 0.12 mmol/L vs. TD4).

### 3.2. Changes in Serum Insulin, HOMA-IR and Angiotensin Converting Enzyme of HFD-Fed Rats

**Figure 2:**
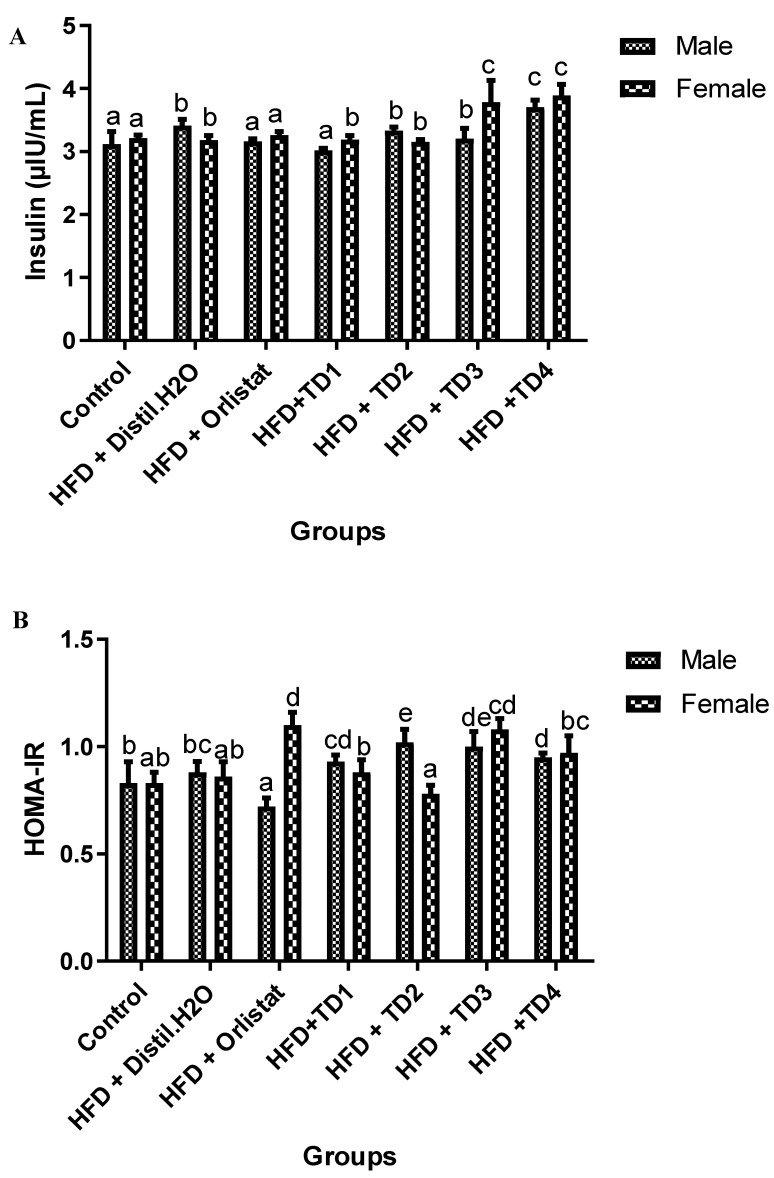

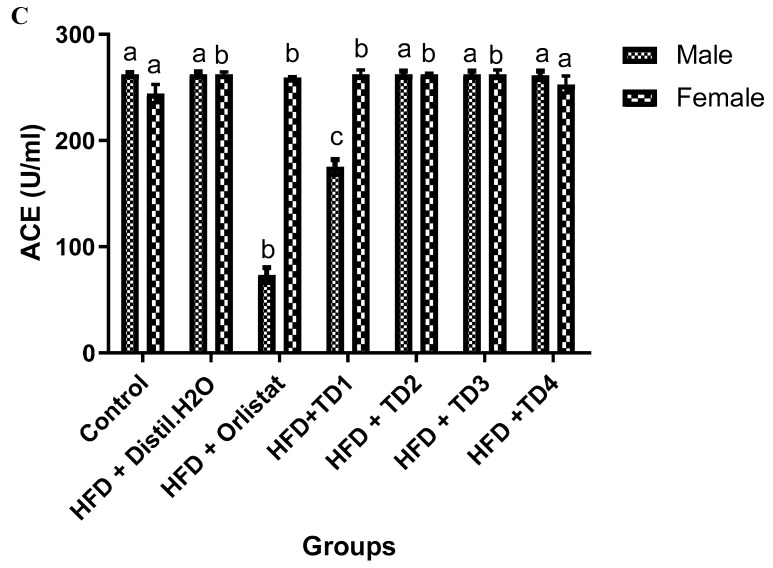
Effect of dietary interventions with HFD on (**A**) fasting insulin, (**B**) HOMA-IR, (**C**) Angiotensin converting enzyme concentration. Values were expressed as mean ± SEM (*n* = 7). Mean values with different superscript letters within the same column of the sex group are significantly different at *p* < 0.05.

To further evaluate the effect on glucose metabolism, fasting serum insulin levels were measured in both male and female rats after 10 weeks of dietary interventions, which included the HFD alone or supplemented with TD formulations (TD1–TD4) or Orlistat (Figure 2A). Baseline control rats exhibited insulin levels of 3.12 ± 0.20 µIU/mL in males and 3.21 ± 0.05 µIU/mL in females, reflecting normal insulin homeostasis. HFD supplementation alone slightly increased insulin concentrations in males but showed a minor decrease in females, suggesting a mild sex-specific difference in response to HFD. Interestingly, co-supplementation with TD compounds resulted in different insulin patterns. In the HFD + TD1 group, male insulin concentrations remained comparable to control values, suggesting a minimal impact on basal insulin. In contrast, HFD + TD2 led to modest increases, although differences remained statistically insignificant. Notably, HFD + TD3 and HFD + TD4 significantly elevated insulin concentrations in both sexes (*p* < 0.05 vs. control). These results indicate that TD3 and TD4 induced a marked hyperinsulinemic response, potentially reflecting enhanced pancreatic insulin secretion or compensatory mechanisms associated with HFD-induced insulin resistance.

Insulin resistance indices were further assessed using the HOMA-IR across experimental groups (Figure 2B). Control rats displayed similar HOMA-IR values in both sexes, indicating normal insulin sensitivity. HFD supplementation slightly increased HOMA-IR in male and female rats, consistent with early signs of insulin resistance. Among the TD-treated groups, TD1 exhibited HOMA-IR values comparable to those of the HFD control in both males and females. TD2 elevated HOMA-IR in males but reduced it in females, again suggesting sex-specific modulation. In contrast, TD3 resulted in increased HOMA-IR in both sexes, consistent with the hyperinsulinemic effect observed in insulin measurements.

The effects of HFD and subsequent interventions on serum ACE concentration are shown in Figure 2C. In the control group, ACE levels were relatively stable in both sexes, with values of 262.33 ± 1.7 U/L in males and 244.12 ± 8.4 U/L in females. Administration of HFD alone did not significantly alter ACE activity compared to controls, with values in both male and female remaining at approximately 262.33 U/L. This suggests that HFD feeding, within the duration of this experiment, did not markedly influence circulating ACE concentrations.

Among the TD formulations, only TD1 had a significant effect, particularly in male rats. ACE concentration in males decreased to 175 ± 7.03 U/L following TD1 supplementation, while female ACE levels remained unchanged relative to controls. This partial reduction in males demonstrates a modest ACE-inhibitory effect of TD1, though less potent than Orlistat. TD2 and TD3 treatments had no significant impact on ACE concentration in either sex, with values remaining similar to those of HFD and control groups (approximately 262.33 U/L). Likewise, TD4 showed minimal influence, with male and female values (261.33 ± 4.04 and 252.58 ± 8.02 U/L, respectively) closely relative the control levels.

### 3.3. Changes in Systemic Lipid Profiles

To evaluate the systemic metabolic impact of the HFD and the dietary intervention, serum lipid parameters were measured (Table 1). Overall, lipid profile evaluation demonstrated that HFD feeding induced pronounced dyslipidemia, with distinct treatment responses and sex-specific patterns. In males, HFD significantly increased circulating triglycerides and concomitantly reduced HDL relative to controls. Supplementation of TD1 and TD2 significantly reduced LDL concentrations, indicating beneficial modulation of lipoprotein metabolism. TD3 markedly reduced triglycerides (*p* < 0.01) and improved HDL (*p* < 0.05), indicating strong anti-hyperlipidemic and cardioprotective potential. In contrast, TD4 exerted a more complex influence, significantly lowering LDL (*p* < 0.05) but simultaneously increasing triglycerides and total cholesterol, indicating a divergent regulatory profile.

In females, the lipid response diverged markedly from that observed in males. Orlistat significantly increased triglyceride concentrations (5.10 ± 0.48 mmol/L; *p* < 0.05) while reducing HDL (3.05 ± 0.27 mmol/L; *p* < 0.05), which contrasts with its documented lipase-inhibiting effects. This anomaly may reflect sex-specific metabolic responses, differences in dietary fat composition, or the short intervention duration. TD1 and TD2 were the most favorable interventions, producing significant increases in HDL while maintaining total cholesterol and LDL at moderate levels, suggesting protective cardiometabolic potential. TD3 yielded the lowest LDL concentrations but was also associated with a profound reduction in HDL, which limited its therapeutic benefit. TD4 significantly reduced total cholesterol but concomitantly decreased HDL, indicating a mixed effect on lipid regulation. These sex-dependent differences in lipid regulation may be attributed to variations in lipid metabolism, adipose tissue distribution, and hormonal influences, which are known to modify lipid handling and the response to bioactive compounds [22].

### 3.4. Response of Composite 3D-Printed Snacks to Serum Sex Hormones

**Figure 3:**
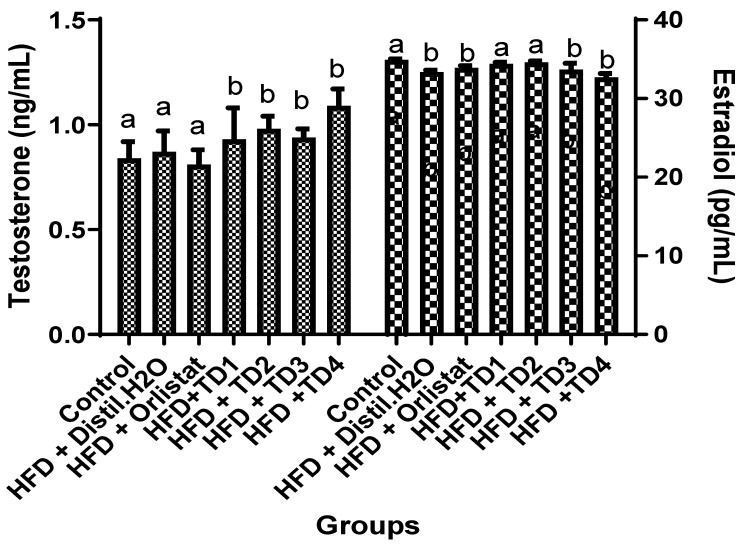
Serum sex hormone responses to composite 3D-printed snacks supplementation in rats. Values were expressed as mean ± SEM (*n* = 7). Mean values with different superscripts within the same column of the sex group are significantly different at *p* < 0.05.

Given the observed sex-specific responses in previous analyses, this experiment was conducted to evaluate the effects of composite 3D-printed snacks on circulating concentrations of key sex hormones, including testosterone in male and estrogen in female rats (Figure 3). Serum sex hormone analysis revealed distinct sex-dependent responses following supplementation with composite 3D-printed snacks. In male rats, supplementation with TD1-TD4 significantly increased testosterone concentrations relative to both control and HFD groups (*p* < 0.05), with TD4 producing the highest increase (1.09 ± 0.08 ng/mL), representing approximately a 30% elevation above control. In female rats, HFD feeding reduced estradiol concentrations compared with controls. Supplementation with TD1 and TD2 restored estradiol levels to values comparable to those of control animals, indicating a protective effect against HFD-induced hormonal disruption. In contrast, TD3 and, particularly, TD4 (32.67 ± 0.50 pg/mL) resulted in significant decreases in estradiol compared to the controls. These findings demonstrate pronounced sex-specific hormonal responses to the composite snacks. TD2 exerted the most balanced effect, enhancing testosterone in males while maintaining estradiol within normal ranges in females. Conversely, TD4 markedly elevated testosterone in males but suppressed estradiol in females, highlighting a clear sex-specific divergence in hormonal regulation induced by the composite snacks.

### 3.5. Histological Examination of Heart Tissues

**Figure 4:**
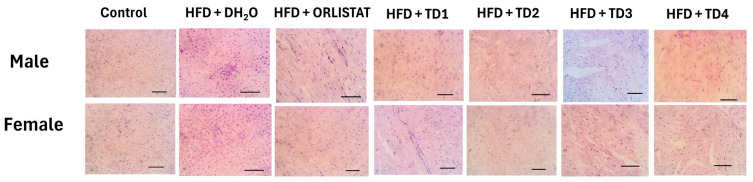
Effect of composite 3D printed snacks on the heart histology of the high-fat diet Wistar rats. H&E Stain. Scale bar = 20 µm.

Hematoxylin and eosin staining was used to assess cardiac architecture in male and female rats across all experimental groups (Figure 4). In both sexes, an inflammatory reaction was observed around the portal area in the HFD + DH_2_O group, indicating HFD-induced cardiac stress. In contrast, all groups supplemented with the composite 3D-printed snacks displayed preserved myocardial structure. Myocardial fibers remained intact with no evidence of inflammation or fibrosis. These findings indicate that the dietary supplementation with composite 3D-printed snacks did not induce lesions or structural alterations in heart tissue.

## 4. Discussion

The present study investigated the potential of bioactive-enriched 3D-printed snacks, formulated from germinated and probiotic-fermented flours, to mitigate HFD-induced metabolic dysfunction in Wistar rats. Collectively, the findings demonstrate that these novel functional foods exerted significant improvements in glycemic control and lipid metabolism, albeit with distinct treatment and sex-specific responses. Consistent with previous studies demonstrating that HFD causes high blood sugar and early insulin resistance [5,23], our results showed significant increases in fasting blood glucose and HOMA-IR in both sexes after HFD induction. Supplementation with 3D-printed snacks, particularly TD4, significantly reduced fasting blood glucose levels in male and female rats, restoring glycemic values to near baseline values (Figure 1). This is consistent with evidence that fermented legume- and cereal-based products enhance glycemic control through increased bioavailability of bioactive peptides and phenolic compounds [8]. However, the strong increase in insulin levels induced by TD3 and TD4 suggests the pancreas is working harder to keep glucose under control, which could lead to β-cell stress if this continues long-term [24]. In contrast, TD1 maintained near-normal insulin and HOMA-IR values, indicating a more favorable stabilization of glucose–insulin dynamics without excessive compensatory secretion [25].

HFD feeding caused dyslipidemia in both sexes, with higher triglycerides and lower HDL cholesterol, a pattern linked to atherosclerosis and heart disease [22,26]. Intervention with the composite 3D-printed snacks produced different effects in males and females. In males, TD3 significantly reduced triglyceride concentrations while increasing HDL, suggesting potent anti-hyperlipidemic and cardioprotective effects (Table 1). Mechanistically, this may reflect the role of probiotic fermentation in generating short-chain fatty acids (SCFAs), such as butyrate and propionate, which are known to regulate hepatic lipid metabolism and promote HDL biogenesis [12]. In females, the lipid responses diverged significantly. TD1 and TD2 prompted favorable increases in HDL while maintaining LDL and total cholesterol at moderate levels, demonstrating a cardioprotective lipid profile. These findings resonate with previous reports that estrogen status interacts with dietary bioactives to modulate lipid handling, where estrogen tends to favor higher HDL concentrations [27,28]. Conversely, TD3, despite lowering LDL, markedly reduced HDL, thus offsetting its cardiometabolic benefit. These sex-specific patterns likely reflect both compositional differences in the 3D snacks and inherent biological differences in lipid metabolism between males and females.

A novel aspect of this study was the examination of reproductive hormone modulation by the HFD and dietary interventions. Testosterone concentrations in males were significantly elevated by TD2 and TD4, suggesting stimulatory properties of the bioactive compounds in these formulations. Conversely, estradiol levels in females were preserved by TD1 and TD2 but suppressed by TD3 and TD4, suggesting possible disruption of ovarian hormone regulation (Figure 3). This confirms sex-specific hormonal effects, similar to reports showing that dietary bioactives affect male and female reproductive hormones differently [29]. The divergent responses observed may also be attributed to well-established biological differences in hormonal regulation, adipose tissue distribution, and metabolic signaling. For example, estrogen enhances HDL production, improves insulin sensitivity, and promotes anti-inflammatory pathways in females, whereas testosterone modulates lipid turnover, increases lean mass, and influences insulin action through androgen-receptor–mediated mechanisms in males [30,31]. More recent work also demonstrates that estradiol and testosterone differentially regulate mitochondrial activity, adipocyte function, and glucose utilization, contributing to sex-specific adaptive patterns under high-fat feeding [32]. These mechanisms provide a biological basis for the sex-dependent differences observed in glycemic control, lipid regulation, ACE activity, and hormone profiles in this study, underscoring the importance of considering sex-specific formulations in the development of functional foods for metabolic health.

Beyond metabolic and hormonal parameters, TD1 significantly reduced serum ACE concentrations in males, while other diets had minimal effects. ACE, a central component of the renin–angiotensin system, links cardiovascular regulation with metabolic disorders. Its activity is upregulated in adipose tissue, contributing to adipocyte hypertrophy, inflammation, and insulin resistance. This aberrant activation of the ACE–angiotensin II axis promotes oxidative stress and inflammation by increasing reactive oxygen species and proinflammatory mediators [33]. TD1’s ACE-inhibitory activity suggests potential downregulation of the renin–angiotensin system, offering an additional cardioprotective mechanism. Histological analyses further supported these findings; the HFD group exhibited perivascular inflammatory cell infiltration in both sexes, consistent with low-grade systemic inflammation induced by HFD [5]. All groups (TD1–TD4) maintained normal myocardial architecture, as there was no evidence of necrosis, inflammatory infiltration, or fibrosis (Figure 4), demonstrating that the composite 3D-printed snacks did not alter cardiac morphology in either sex, reinforcing their potential as safe dietary interventions for obesity-associated metabolic dysfunction.

The literature on 3D food printing emphasizes its potential for personalization and functional enhancement [18,34,35], but evidence of in vivo benefits remains scarce. In this study, 3D-printed snacks derived from germinated and probiotic-fermented flours show strong potential as functional foods for managing obesity. Their ability to improve glycemic control, modulate lipid profiles, and influence endocrine and cardiovascular pathways highlights their translational value. However, the observed sex-specific differences highlight the necessity of precision formulation strategies, which 3D food printing is uniquely equipped to deliver.

## 5. Conclusions

This study demonstrates that both raw and bioprocessed 3D-printed snacks formulated from indigenous composite inks of cowpea, sorghum, and orange-fleshed sweet potato improved several obesity-related outcomes in Wistar rats. The interventions produced distinct, formulation- and sex-specific responses; TD4 produced the strongest improvement in glycemic control, while TD3 significantly reduced triglycerides and increased HDL in males. In females, TD1 and TD2 enhanced HDL and maintained a favorable lipid profile. Moreover, only TD1 reduced ACE activity in males, suggesting a unique cardioprotective effect not observed with the other formulations. These outcomes reveal clear sex-specific patterns in metabolic and hormonal regulation, emphasizing that although the snacks effectively attenuate high-fat-diet-induced dysfunctions, their benefits vary according to processing state, blend ratio, and biological sex. Collectively, the findings highlight the potential of 3D-printed functional foods as a safe and innovative strategy for obesity management, with promising opportunities for precision nutrition tailored to sex-specific needs. Further work could explore longer study periods, assessing other parameters, including inflammation markers, oxidative stress, gut integrity, behavioral effects, and the impact on other organs and tissues.

## Figures and Tables

**Table 1:** Lipid profile for male and female rats.

Groups	Triglyceride (mmol/L)	High-Density Lipoprotein (mmol/L)	Total Cholesterol (mmol/L)	Low-Density Lipoprotein (mmol/L)
Male				
Control	1.57 ± 0.40 ^b^	6.82 ± 0.13 ^g^	5.04 ± 0.10 ^a^	4.72 ± 0.07 ^a^
HFD + Distilled H_2_O	2.14 ± 0.08 ^d^	3.99 ± 0.36 ^e^	4.40 ± 0.08 ᵈ	4.99 ± 0.45 ^a^
HFD + Orlistat	2.02 ± 0.10 ^c^	3.49 ± 0.68 ^b^	3.32 ± 0.35 ^c^	4.36 ± 0.00 ^a^
HFD + TD1	2.78 ± 0.72 ^f^	2.64 ± 0.54 ^a^	3.42 ± 0.39 ^c^	3.13 ± 0.54 ^c^
HFD + TD2	2.33 ± 0.35 ^e^	3.68 ± 0.10 ^d^	4.15 ± 0.10 ^c^	3.00 ± 0.25 ^c^
HFD + TD3	1.12 ± 0.22 ^a^	4.52 ± 0.66 ^f^	3.58 ± 0.74 ^c^	3.94 ± 0.00 ᵇ
HFD + TD4	3.04 ± 0.73 ^g^	3.59 ± 0.42 ^c^	5.63 ± 0.66 ᵇ	2.27 ± 0.23 ^c^
Female				
Control	1.82 ± 0.07 ^a^	4.49 ± 0.07 ^a^	5.37 ± 0.03 ^a^	7.10 ± 0.69 ^a^
HFD + Distilled H_2_O	1.93 ± 0.05 ^a^	4.82 ± 0.82 ᵇ	2.53 ± 0.57 ᵇ	4.46 ± 0.08 ^c^
HFD + Orlistat	5.10 ± 0.48 ^c^	3.05 ± 0.27 ᶠ	4.59 ± 1.09 ^c^	4.29 ± 0.00 ^c^
HFD + TD1	1.95 ± 0.57 ᵇ	7.87 ± 1.07 ᵈ	4.57 ± 0.69 ^c^	5.25 ± 0.34 ^c^
HFD + TD2	1.32 ± 0.45 ^a^	5.48 ± 0.44 ^c^	4.89 ± 0.33 ^c^	4.61 ± 0.00 ^c^
HFD + TD3	1.86 ± 0.00 ^a^	1.42 ± 0.21 ^c^	3.96 ± 0.72 ᵇ	2.59 ± 0.12 ᵇ
HFD + TD4	2.14 ± 0.34 ᵇ	2.95 ± 0.00 ^c^	3.08 ± 0.68 ᵇ	4.61 ± 0.29 ^c^

Values were expressed as mean ± SEM (*n* = 7). Mean values with different superscripts within the same column of the sex group are significantly different at *p* < 0.05.

## Data Availability

The original contributions presented in this study are included in the article. Further inquiries can be directed to the corresponding author.
